# Non-alcoholic fatty liver disease phenotypes in patients with inflammatory bowel disease

**DOI:** 10.1038/s41419-017-0124-2

**Published:** 2018-01-24

**Authors:** Alessandro Sartini, Stefano Gitto, Marcello Bianchini, Maria Chiara Verga, Maria Di Girolamo, Angela Bertani, Mariagrazia Del Buono, Filippo Schepis, Barbara Lei, Nicola De Maria, Erica Villa

**Affiliations:** Department of Internal Medicine, Gastroenterology Unit, University of Modena and Reggio Emilia, Modena, Italy

## Abstract

Non-alcoholic fatty liver disease (NAFLD) can be detected in up to 33.6% of inflammatory bowel disease (IBD) patients, often in absence of metabolic risk factors. Nevertheless, most of previous studies on such issue were conducted within the IBD population only. The primary aim of this study was to compare clinical and metabolic features of NAFLD in patients with and without IBD (w/o IBD) and to identify specific NAFLD phenotypes within the IBD population. Among 223 NAFLD patients, 78 patients with IBD were younger compared to 145 without (w/o) IBD, were less likely to have altered liver enzymes, had lower mean body weight, smaller waist circumference and lower body mass index (BMI); at the same time, MetS was more prevalent among patients w/o IBD (56.6 vs. 23.1%, *p* < 0.001). Within IBD population, patients with severe IBD showed more often severe steatosis (S3) at ultrasound (US) (32.1 vs. 16.6%, *p* = 0.01), compared to mild-to-moderate disease. Independent risk factors for S3 US steatosis in IBD patients at the multivariate logistic regression analysis were: more than 1 IBD relapse per year during disease history (OR 17.3, 95% CI 3.6–84), surgery for IBD (OR 15.1, 95% CI 3.1–73.7) and more extensive intestinal involvement (OR 19.4, 95% CI 3.4–110.9); the ongoing anti-Tumor Necrosis Factor alpha (antiTNFα) therapy was the only independent factor which protect toward the presence of altered liver enzymes (OR 0.15, 95% CI 0–0.8, *p* = 0.02). In conclusion, NAFLD in IBD patients is different from that in patients w/o IBD, who seem to develop different NAFLD phenotypes according to intestinal disease clinical course. More severe IBD seem to predict the presence of more severe steatosis. Therapy with antiTNFα antibodies could prevent alteration of liver enzymes in such population.

## Introduction

Non-alcoholic fatty liver disease (NAFLD) includes a wide spectrum of disorders, ranging from hepatic steatosis (NAFL) to non-alcoholic steatohepatitis (NASH). NAFLD patients are at high risk for liver fibrosis, cirrhosis, and hepatocellular carcinoma (HCC)^1–5^. Although NAFLD is typically associated with altered metabolism and metabolic syndrome (MetS), it also occurs in patients with inflammatory bowel disease (IBD). In this context, NAFLD is usually considered the consequence of malnutrition and malabsorption^6^.

The prevalence of NAFLD in IBD patients is highly variable ranging from 1.5% to even 40%, in dependence of different diagnostic criteria^7–9^. In a recent retrospective study, Bessissow et al.^10^ confirmed that NAFLD is often diagnosed in IBD patients (prevalence 33,6%, incidence rate 9.1/100 PY); at baseline, IBD patients who developed NAFLD during the observation period were older, more often diabetics and with a higher average body mass index (BMI) than those who did not develop NAFLD^10^.

Nevertheless, prevalence of obesity and diabetes in such population was low and some Authors hypothesized that the pathogenesis of NAFLD in IBD patients could involve disease-specific risk factors, related, for example, to the underlying chronic inflammatory status^11,12^. In this view, disease activity and duration, steroid use during the inflammatory bowel disease progression, small bowel surgery and alterations of the gut microbiota, were identified as predictors of NAFLD in such patients; these factors could be considered as surrogate markers of IBD severity.

Taken together, these data suggest that IBD patients could develop NAFLD with less metabolic risk factors than the general population^10,11,13^. However, most studies on this issue were conducted in IBD patients only, without a direct comparison of clinical features and natural history of NAFLD in non-IBD patients^1,4,14,15^. Moreover, the impact of NAFLD-related features on the progression of IBD is completely unknown.

The primary aim of this retrospective study was to characterize NAFLD in patients with and without IBD (w/o IBD), comparing their clinical and metabolic features. According to the presence of severe steatosis and altered liver enzymes, the secondary aim of the study was to identify specific NAFLD-phenotypes within the IBD population, relating them to the course of the intestinal disease.

## Results

### Study chart

**Fig. 1:**
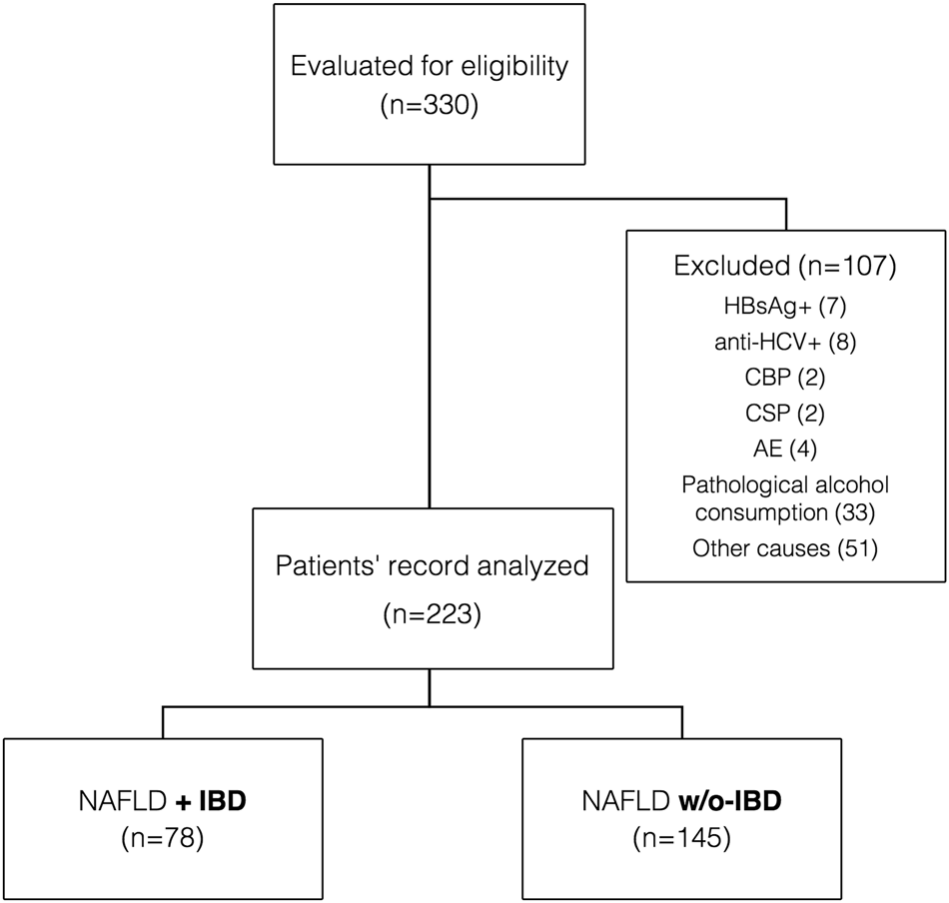
Study flow chart

Three-hundred and 30 consecutive patients were evaluated for study eligibility, 107 of whom were excluded because they did not meet the exclusion criteria (Fig. 1). The demographic and clinical data of the remaining 223 patients, 78 NAFLD + IBD and 145 NAFLD without (w/o) IBD were collected and retrospectively analyzed.

### Clinical and metabolic characteristics of NAFLD + IBD patients

Clinical and demographic data of the 78 IBD patients at the time of observation are reported in Table 1. All of the 78 IBD patients had NAFLD diagnosis prior to IBD diagnosis. Forty-two out of 78 patients with NAFLD + IBD had Crohn’s disease (53.8%) and 36 (46.2%) had Ulcerative colitis. CD and UC patients did not significantly differ for clinical and metabolic characteristics. Forty-three out of 78 patients (55.1%) had extensive IBD and 26 patients (33.3%) had clinical and/or endoscopic-documented active disease lasting at least 3 months. Thirty-four out of 78 patients (43.6%) had history of more than 1 disease relapse/year since IBD diagnosis. Twenty-seven out of patients 78 (34.6%) had had surgery during their IBD course (ileal or ileo-colonic resection for 20 CD patients, none of whom developed short bowel syndrome and total subtotal or total procto-colectomy with ileo-anal anastomosis for 7 UC patients). Concerning therapies, and particularly corticosteroids (CCSs), the majority of patients with more than 1 disease flare/year (25 out of 34, 73.5%) had received only low-systemic bioavailable corticosteroids (budesonide or beclomethasone per os or enema) for relapse treatment during their IBD course, while the remaining 9 patients had received systemic CCSs. Seven patients (8.9%) had been on low-systemic bioavailable steroids therapy for at least the previous 3 months before the first detection of steatosis and only 3 patients were taking budesonide at the time of steatosis detection. Ongoing medications are detailed in Table 1.

**Table 1:** Demographic characteristics of studied patients

Variables	NAFLD + IBD patients (*n* = 78)
Age at IBD diagnosis, mean ± SD, yr	43.32 ± 13.48
Crohn’s disease, *n* (%)	42 (53.8)
*Location*	
• Ileal (L1)	19 (45.2)
• Colonic (L2)	5 (11.9)
• Ileo-colonic (L3)	18 (42.9)
Ulcerative Colitis, *n* (%)	36 (46.2)
*Location*	
• Proctitis (E1)	7 (19.4)
• Left-sided (E2)	19 (52.8)
• Pancolitis (E3)	10 (27.8)
Extensive disease, *n* (%)	43 (55.1)
Active disease, *n* (%)	26 (33.3)
More than 1 relapse/year, *n* (%)	34 (43.6)
IBD duration, mean ± SD, months	99.9 ± 91.1
Surgery for IBD, *n* (%)	27 (34.6%)
Ongoing medications, *n* (%)	
5ASA	51 (65.4)
• CCS	3 (3.8)
• CCS + 5ASA	4 (5.1)
• AZA/6MP	4 (5.1)
• antiTNFα	15 (19.2)
• Other	1 (1.3)

*SD* standard deviation, *5ASA* Mesalamine, *CCS* Corticosteroids, *AZA* Azathioprine, *6MP* 6-Mercaptopurine, *antiTNFα* anti-Tumor Necrosis Factor α antibodies

Concerning the metabolic profile, MetS was more prevalent in patients who had stable clinical and endoscopic remission for more than 6 months than in those with active disease (30.8 vs. 7.7%, *χ*^2^-square test *p* = 0.02). Patients in clinical stable remission for more than 6 months had higher mean BMI compared to patients with clinical activity in the previous 3 months (27.9 ± 4.5 Kg/m^2^ vs. 25.5 ± 4.9 Kg/m^2^, *p* = 0.04).

### Comparison of demographic and metabolic data of patients with NAFLD and IBD vs. NAFLD without IBD

NAFLD patients with IBD were younger compared to NAFLD w/o IBD (mean ± SD 51.2 ± 11.8 vs. 54.9 ± 12.5 years, *p* = 0.03) and were less likely to have altered liver enzymes (42.3 vs. 57.9%, *p* = 0.03). Mean white blood cells (WBCs), platelets (PLTs) and mean C-reactive protein (CRP) were higher in IBD patients (*p* < 0.001, *p* = 0.001, and *p* = 0.001, respectively) likely because of patients with active disease within IBD group (Table 2).

**Table 2:** Comparison of clinical and demographic characteristics of NAFLD patients with and without IBD

Variables	NAFLD + IBD (*N* = 78)	NAFLD w/o IBD (*N* = 145)	*p*-value
Age, mean ± SD, yr	51.19 ± 11.82	54.88 ± 12.50	**0.03** ^1^
Male gender, *n* (%)	49 (62.8%)	43 (29.7%)	**<0.01** ^2^
AST, mean ± SD, IU/L	31.70 ± 13.91	38.99 ± 25.86	**0.01** ^1^
ALT, mean ± SD, IU/L	42.28 ± 28.81	53.83 ± 45.01	**0.03** ^1^
γGT mean ± SD, IU/L	68.96 ± 64.89	61.77 ± 66.00	0.43^1^
WBC, mean ± SD, *n* × 10^3^	9.51 ± 3.24	6.93 ± 1.87	**<0.01** ^1^
Platelets, mean ± SD, *n* × 10^3^	301.15 ± 133.41	236.83 ± 74.63	**<0.01** ^1^
CRP, mean ± SD, mg/L	22.2 ± 36.2	4 ± 7.1	**<0.01** ^1^
US steatosis grading			
• Mild-to-moderate (S1 + S2)	53 (67.9%)	121 (83.4%)	**0.01** ^2^
• Severe (S3)	25 (32.1%)	25 (16.6%)	
Stiffness, mean ± SD, kPa	5.2 ± 1.69	6.4 ± 3.52	0.09^1^
APRI score, mean ± SD	0.46 ± 0.34	0.63 ± 0.52	0.09^1^
Splenic BPD, mean ± SD, cm	10.76 ± 1.83	10.65 ± 2.21	0.74^1^
Altered liver enzymes, *n* (%)	33 (42.3%)	84 (57.9%)	**0.03** ^2^

*SD* standard deviation, *AST* aspartate aminotransferase, *ALT* alanine aminotransferase, *γGT* gamma glutamiltransferase, *WBC* white blood cells, *CRP* C-reactive protein, *BDP* bipoplar diameter

^1^Student’s *t*-test

^2^Pearson’s *χ*^2^-test or two-tailed Fisher’s exact

Analyzing the clinical and metabolic profile, IBD patients had lower mean (±SD) body weight (78.1 ± 13.3 Kg vs. 82. ± 15.8 Kg, *p* = 0.04), smaller mean waist circumference (97.7 ± 14 cm vs. 102 ± 14.5 cm, *p* = 0.04) and lower BMI (28.6 ± 4.8 Kg/m^2^ vs. 30.2 ± 5.4 Kg/m^2^ *p* < 0.001); at the same time, they less often had hypertension, reduced HDL cholesterol level and elevated fasting plasma glucose. Mean total/LDL/HDL cholesterol and triglycerides did not significantly differ between the two groups. On the whole, MetS was more prevalent among patients w/o IBD (56.6 vs. 23.1%, *p* < 0.001) (Table 3).

**Table 3:** Comparison of metabolic profile in NAFLD patients with and without IBD

Variables	NAFLD + IBD (*N* = 78)	NAFLD w/o IBD (*N* = 145)	*p*-value
Weight, mean ± SD, Kg	78.1 ± 13.3	82.3 ± 15.8	**0.04** ^1^
Waist circumference, mean ± SD, cm	97.7 ± 14	102 ± 14.5	**0.04** ^1^
BMI, mean ± SD, Kg/m^2^	28.63 ± 4.83	30.22 ± 5.38	**<0.001** ^1^
Obesity, *n* (%)	21 (26.9%)	72 (52.4%)	**<0.001** ^2^
Total cholesterol, mean ± SD, mg/dL	214.25 ± 42.99	218.01 ± 43.73	0.8^1^
LDL cholesterol, mean ± SD, mg/dL	133.94 ± 37.1	130.24 ± 37.9	0.55^1^
HDL cholesterol, mean ± SD, mg/dL	51.02 ± 14.54	49.35 ± 15.40	0.48^1^
Triglycerides, mean ± SD, mg/dL	167.41 ± 88.64	143.3 ± 66.7	0.92^1^
Elevated TG, *n* (%)	22 (29.5%)	53 (36.6%)	0.28^1^
Fasting plasma glucose, mean ± SD, mg/dL	92.67 ± 14.82	108.2 ± 32.7	0.16^1^
Elevated glucose, *n* (%)	23 (29.5%)	65 (44.8%)	**0.03** ^2^
Insulin, mean ± SD, µIU/mL	15.92 ± 11.72	12.65 ± 6.92	0.24^1^
Hypertension, *n* (%)	19 (24.4%)	76 (52.4%)	**<0.001** ^2^
Reduced HDL cholesterol, *n* (%)	20 (31.2%)	67 (48.9%)	**0.02** ^2^
Metabolic syndrome, *n* (%)	18 (23.1%)	82 (56.6%)	**<0.001** ^2^

*SD* standard deviation, *BMI* body mass index, *LDL* low-density level, *HDL* high-density level, *TG* triglycerides

^1^Student’s *t*-test

^2^Pearson’s *χ*^2^-test or two-tailed Fisher’s exact

### Risk factors for severe steatosis in IBD patients

NAFLD patients with severe IBD showed significantly more often severe steatosis at US (grading S3) at the time of the first detection of steatosis (32.1 vs. 16.6%, *p* = 0.01). Stiffness measurement and APRI score did not significantly differ between the 2 groups.

To identify risk factors for severe (S3) US steatosis in IBD patients, a binary logistic regression analysis considering intestinal-related and metabolic-related variables was performed. Both at univariate and multivariate analysis, risk factors for severe US steatosis (grading S3) were: more than 1 IBD relapse per year during disease history (OR 17.3, 95% CI 3.6–84, *p* < 0.001), surgery for IBD (OR 15.1, 95% CI 3.1–73.7, *p* = 0.001), and more extensive intestinal involvement (OR 19.4, 95% CI 3.4–110.9, *p* = 0.001). The diagnosis of MetS did not significantly relate with severe grading of steatosis within such subgroup of patients (Table 4).

**Table 4:** Risk factors for S3 steatosis among IBD patients

Variables	Univariate		Multivariate	
	Odds ratio (95% CI)	*p*-value	Odds ratio (95% CI)	*p*-value
>1 relapse/yr	8 (2.7–24)	**<0.001**	17.3 (3.6–84)	**<0.001**
Surgery for IBD	6.8 (2.4–19.4)	**<0.001**	15.1 (3.1–73.7)	**0.001**
Extensive IBD	7.4 (2.2–24.6)	**0.001**	19.4 (3.4–110.9)	**0.001**
Metabolic syndrome	1.5 (0.5–4.5)	0.48	—	—

### Risk factors for altered liver enzymes in NAFLD + IBD patients

Concerning the presence of altered liver enzymes (AST/ALT), the most representative factors of the intestinal and hepatic conditions were included in the regression logistic model (Table 5).

**Table 5:** Risk factors for altered liver enzymes among IBD patients

Variables	Univariate		Multivariate	
	Odds ratio (95% CI)	*p*-value	Odds ratio (95% CI)	*p*-value
Severe US steatosis (S3)	3.5 (1.3–9.3)	**0.01**	2.7 (0.9–8.3)	0.08
Ongoing antiTNFα	0.2 (0–0.8)	**0.03**	0.15 (0–0.8)	**0.02**
Extensive IBD	3.2 (1.2–8.1)	**0.02**	2.3 (0.8–6.7)	0.12
Metabolic syndrome	0.8 (0.3–2.3)	0.6	—	—

At univariate analysis, severe steatosis (S3) (OR 3.5, 95% CI 1.3–9.3, *p* = 0.01) and more extensive IBD (OR 3.2, 95% CI 1.2–8.1, *p* = 0.02) were risk factors for increased AST/ALT, while the ongoing therapy with anti-Tumor Necrosis Factor alpha (antiTNFɑ) antibodies showed a protective effect (OR 0.2, 95% CI 0–0–8, *p* = 0.03). At multivariate analysis, only ongoing antiTNFɑ therapy was an independent protective factor for the presence of altered liver enzymes (OR 0.15, 95% CI 0–0.8, *p* = 0.02).

## Discussion

In this study, we compared NAFLD phenotype in patients with and without IBD and we identified factors associated, in the IBD population, with the presence of severe steatosis and with increased AST/ALT levels: these are potential determinants for progression of liver condition toward liver fibrosis and cirrhosis^3–5,16^.

Although NAFLD is typically related to MetS and dysregulated metabolism, it can be found also in IBD patients, which generally have lower BMI and lower prevalence of metabolic risk factors^6^. As mentioned above, a large study conducted on 928 IBD patients identified small bowel surgery (OR 3.7), hypertension (OR 3.5), obesity (OR 2.1), and the use of CCSs at the time of US examination (OR 3.7) as independent risk factors for presence of NAFLD in patients with IBD^7^. On the contrary, in our study CCS therapy did not predict the presence of severe steatosis among IBD patients. Systemic CCSs notably cause liver steatosis by inhibiting mitochondrial beta-oxidation and lipid beta-peroxidation enzymes. However, the majority of IBD patients in our study had received only low-systemic bioavailable CCSs, such as budesonide, which have an extensive first pass liver metabolism.

McGowan et al.^6^ had previously identified in the improvement of therapies and nutritional status the cause of increased BMI among IBD population, with a direct relationship between prolonged remission, higher risk of MetS and NAFLD development. More recently, Erzin et al.^17^ did not find any difference between prevalence of NAFLD in IBD and Irritable Bowel syndrome (IBS) patients, assuming that the pathogenesis of steatosis and steatohepatitis in patients with IBD was related more to nutritional factors than to inflammatory load. Still, IBD activity and its severity have been recently linked to NAFLD development^18–21^: Bessissow et al. found that, after adjusting for steroids treatment, age at IBD diagnosis and use of antiTNFɑ drugs or methotrexate, the development of NAFLD was independently predicted by the presence of active IBD (HR 1.58), disease duration (HR 1.12) and prior bowel surgery (HR 1.34), all surrogate markers of severe disease^10^. None of these Authors, however, compared NAFLD phenotype in patients with and without IBD; this could help to better identify specific features of NAFLD within IBD population and to find predictors of bowel disease severity. In our study, patients with NAFLD and IBD were younger than those w/o IBD, had lower BMI and lower prevalence of MetS but more often severe steatosis (S3) at US. Overall, these features indicate that NAFLD in IBD patients is notably different from what is observed in patients w/o IBD. According to the severity of intestinal disease, we identified two different specific NAFLD-phenotypes within the IBD population:

1. The “mild” IBD patient, with less than one relapse per year and mild-to-moderate steatosis at the ultrasound (S1, S2), despite having one or more metabolic risk factors, such as increased BMI and/or MetS.
2. The “severe” IBD patient, with more than one relapse/year, more extensive disease, often with a history of bowel surgery, and severe steatosis at ultrasound (S3), but less metabolic risk factors.

In our view, more severe IBD promotes the development of severe liver fat accumulation. Severe liver steatosis further impairs bowel disease. Both IBD and NAFLD are known to be associated to increased intestinal permeability: the translocation of bacterial antigens and DNA throughout the portal system was recently observed in patients with IBD, as well as in those with NAFLD^22,23^. On the same line, of a relevant role played by increased inflammation in NAFLD pathogenesis, is the finding of a protective effect of antiTNFɑ drugs toward severe liver steatosis. Therapy with antiTNFɑ was also the only independent factor positively influencing altered liver enzymes in such subgroup of patients. In a very recent study, Carr et al.^24^ evaluated the influence of MetS on NAFLD severity in patients with IBD. They showed that MetS but not intestinal inflammation predicted NAFLD severity in IBD patients. Particularly, they did not found any significant difference in IBD medication use or in IBD severity. However, they recognized that the majority of IBD patients in their cohort (77%) had NAFLD in the absence of MetS: this suggests that IBD patients develop NAFLD because of an increased inflammatory load and not because of metabolic risk factors.

The lack of histologic diagnosis of NAFLD is a limit of our study: however, qualitative ultrasonography grading of steatosis has already been validated in comparison with histology. Particularly, with a cutoff value >25%, ultrasound was found to have sensitivities and specificities ranging from 85.7 to 99.1% and from 85.2 to 91.9%, respectively, for the detection of moderate or severe hepatic steatosis^25^. Hernaez et al.^26^ confirmed through a meta-analysis that US is an useful tool for diagnosis of both moderate (S2) and severe (S3) steatosis (sensibility and specificity 84.8 and 93.6%, respectively). Considering our patients with severe steatosis, we can therefore assume that this evaluation is an accurate marker of NAFLD. Additionally, the recent guidelines of European Association for the Study of Liver about the management of NAFLD, reported that first-line diagnosis of NAFLD should be done with US^1^. Moreover, we strengthened our study considering only US exams performed into a Third level Center by an expert sonographer and confirming the diagnosis by a second sonographer of exams reporting first-degree (S1) steatosis.

In conclusion, IBD patients seem to develop different NAFLD phenotypes according to intestinal disease clinical course. However, future prospective studies are needed to evaluate whether such specific NAFLD phenotypes could have different prognostic course in this subgroup of patients.

## Patients and methods

### Study design

The study was conducted at a single Centre, the Gastroenterology Unit of the University of Modena and Reggio Emilia, Modena Hospital, Italy. All consecutive patients evaluated at least one time at the NAFLD Outpatient Clinic between March 2012 and March 2016 were assessed for eligibility. Patients with one or more of the following criteria were excluded: lack of complete clinical and demographic parameters; lack of at least one sonographic and one hepatic stiffness evaluation with transient elastography; hepatitis B surface antigen and/or hepatitis C virus antibodies positivity; diagnosis of autoimmune hepatobiliary disease (primary sclerosing cholangitis, primary biliary cholangitis, autoimmune hepatitis), Wilson’s disease or hemochromatosis, according to European Association for the study of Liver (EASL)^27–30^; pathological alcohol consumption, defined as >20 g per day for women and >30 g per day for men^1,31^; diagnosis of coeliac disease. For each patient, demographic and laboratory data, such as previous and ongoing therapies were obtained from clinical reports.

### Diagnostic criteria

Crohn’s disease (CD) and ulcerative colitis (UC) patients were identified among patients with a primary diagnosis of NAFLD according to the European Crohn’s and Colitis Organisation (ECCO) criteria^32,33^; the Crohn’s Disease Activity Index (CDAI) >150 for CD and the Mayo score >2 for UC were used to stratifying IBD activity^34,35^. According to the Montreal classification^36^, we defined disease location for each IBD patient, describing the “extensive disease” as the UC extended beyond the splenic flexure (Montreal E2-E3) and the CD affecting >100 cm in extent, regardless of the location^32^. Ongoing medications were: mesalamine (5ASA) or corticosteroids (CCSs) only, 5ASA + CCSs, azathioprine/6-mercaptopurine (AZA/6-MP), antiTNFɑ antibodies (infliximab or adalimumab) and others (cyclosporine, methotrexate).

The diagnosis of NAFLD was defined according to EASL-EASD-EASO guidelines^1^. Ultrasound imaging of each patient were independently reviewed by two radiologists to grade steatosis (S), classified as mild (S1), moderate (S2), or severe (S3), according to Saverymuttu criteria^37^. All other causes of steatosis were excluded, particularly alcohol abuse over 30 g per day and 20 g per day for men and women respectively and concomitant use of hepatotoxic drugs. The evaluation of liver fibrosis was made according to liver stiffness measurement with transient elastography^38^. The first available ultrasound examination and/or liver stiffness measurement was considered for each IBD and w/o IBD patients, prior to any lifestyle, dietary and/or therapeutic intervention. The APRI score was used to stratify patients for advanced fibrosis^39,40^. Finally, the diagnosis of MetS was based on the Adult Treatment Panel III criteria^41,42^.

### Statistical analysis

Continuous variables were reported as mean (SD) and categorical variables as number of cases and percentage. Student’s *t*-test for independent data or the non-parametric Mann–Whitney *U*-test were used to compare continuous variables; Pearson’s *χ*^2^-test or two-tailed Fisher’s exact test for categorical variables, when appropriate; *p* level <0.05 was considered significant. Univariate and multivariate binary logistic regression analysis was performed considering intestinal-related (number of IBD relapses per year, disease extension, small bowel surgery, and therapies) and metabolic-related variables (diabetes, obesity, and diagnosis of metabolic syndrome) to define predictors of severe steatosis and altered liver enzymes in the subgroup of IBD patients. A *p*-value <0.05 was considered significant for all tests. SPSS software version 23.0 (Chicago, US) was used for statistical analyzes.
